# Rational Management of Iron-Deficiency Anaemia in Inflammatory Bowel Disease

**DOI:** 10.3390/nu10010082

**Published:** 2018-01-13

**Authors:** Ole Haagen Nielsen, Christoffer Soendergaard, Malene Elbaek Vikner, Günter Weiss

**Affiliations:** 1 Department of Gastroenterology, Herlev Hospital, University of Copenhagen, Herlev, DK-2730, Denmark; christoffer.soendergaard@regionh.dk (C.S.); malene.elbaek@mail.dk (M.E.V.); 2 Department of Internal Medicine II, Medical University Hospital of Innsbruck, Innsbruck, A-6020, Austria; Guenter.Weiss@i-med.ac.at; 3 Christian Doppler Laboratory for Iron Metabolism and Anemia Research, University of Innsbruck, Innsbruck, A-6020, Austria.

**Keywords:** anaemia, Crohn’s disease, IBD, iron deficiency, therapy, ulcerative colitis

## Abstract

Anaemia is the most frequent, though often neglected, comorbidity of inflammatory bowel disease (IBD). Here we want to briefly present (1) the burden of anaemia in IBD, (2) its pathophysiology, which mostly arises from bleeding-associated iron deficiency, followed by (3) diagnostic evaluation of anaemia, (4) a balanced overview of the different modes of iron replacement therapy, (5) evidence for their therapeutic efficacy and subsequently, (6) an updated recommendation for the practical management of anaemia in IBD. Following the introduction of various intravenous iron preparations over the last decade, questions persist about when to use these preparations as opposed to traditional and other novel oral iron therapeutic agents. At present, oral iron therapy is generally preferred for patients with quiescent IBD and mild iron-deficiency anaemia. However, in patients with flaring IBD that hampers intestinal iron absorption and in those with inadequate responses to or side effects with oral preparations, intravenous iron supplementation is the therapy of choice, although information on the efficacy of intravenous iron in patients with active IBD and anaemia is scare. Importantly, anaemia in IBD is often multifactorial and a careful diagnostic workup is mandatory for optimized treatment. Nevertheless, limited information is available on optimal therapeutic start and end points for treatment of anaemia. Of note, neither oral nor intravenous therapies seem to exacerbate the clinical course of IBD. However, additional prospective studies are still warranted to determine the optimal therapy in complex conditions such as IBD.

## 1. Introduction

Anaemia and iron deficiency are global health issues and a recent analysis estimated that approximately one-third (i.e., >2.5 billion individuals) of the world’s population is anaemic [1]. Furthermore, it is assumed that more than half the cases of anaemia are caused by an iron-deficient erythropoiesis [1]. Iron deficiency is therefore considered one of the most prevalent global nutritional deficiencies [2]. However, there is a huge geographic variation in prevalence due to a range of sociodemographic factors (i.e., industrialized versus developing countries) [1]. Nevertheless, in addition to anaemia, iron deficiencies cause decrements in energy metabolism, daily activities, quality of life, cognitive and sexual function, cardiac performance and work productivity. However, excess iron can cause cellular oxidative stress and damage by catalysing the formation of toxic radicals via Fenton chemistry [1,2,3,4,5,6].

### 1.1. Anaemia in Inflammatory Bowel Disease 

In inflammatory bowel disease (IBD) [7,8], which shows increasing worldwide incidence and prevalence rates [9,10,11] and which affects up to 0.5% of the population in some countries [12], anaemia is a frequent comorbidity. A recent nationwide Portuguese cross-sectional study of 1287 patients with either Crohn’s disease (CD) (*n* = 775) or ulcerative colitis (UC) (*n* = 512) revealed that flaring disease is the parameter most consistently related to the presence of anaemia, with no differences between CD and UC, although anaemia is more frequent among women [13], especially in CD [14]. Further, an analysis of 171 adult patients with CD showed that iron deficiency was present in 78% with active inflammation but in only 21% with quiescent disease (*p* < 0.001) [15]. It was noticed that markers of CD severity, such as stricturing disease and the need for tumour necrosis factor (TNF) inhibitors and surgery, appeared to be significantly associated with iron deficiency [15]. Moreover, a 2014 systematic review of tertiary referral centres showed a prevalence of anaemia in patients with CD of 27% (95% confidence interval (CI) 19–35%) and in patients with UC of 21% (95% CI 15–27%) [7]. The observed variation mirrored differences in the study populations (e.g., hospitalized patients versus outpatients) as well as the applied definition of anaemia in the studies included. Hence iron deficiency deserves attention in IBD, where the mean prevalence is shown to be 20% among outpatients [16] and 68% among hospitalized patients [17], exceeding by far the frequencies of other extraintestinal manifestations (e.g., rheumatic, dermatologic and ophthalmologic) commonly associated with IBD [18,19].

The pathogenesis of anaemia in IBD is multifactorial and results mainly from intestinal blood loss in inflamed mucosa and impaired dietary iron absorption [20]. The chronic inflammatory state impairs duodenal iron uptake via induction of hepcidin expression in the liver [21] but inflammatory cytokines also have a negative impact on the duodenal uptake of nutrients. Moreover, loss of appetite during flaring disease and a range of other factors such as medications used for IBD treatment (e.g., proton pump inhibitors, sulfasalazine, methotrexate and thiopurines) also have a negative effect on iron absorption and erythropoiesis [22,23]. Vitamin deficiencies, concomitant medical conditions (e.g., renal insufficiency, congestive heart failure, haemolysis, diabetes and innate hemoglobinopathies) [24,25], inflammatory cytokines and acute-phase reactants during flaring disease additionally impair iron availability for erythropoiesis and/or aggravate anaemia by other mechanisms. This blunts the biological response to erythropoietin as well and drives an inflammation-dependent impairment of erythroid progenitor cell proliferation [26,27,28]. Additionally, a predisposition to the development of anaemia may be caused by polymorphisms of iron metabolism genes as well as hormonal factors [29,30,31]. In essence, if the absorptive capacity of iron from the diet does not meet the body’s requirement, iron deficiency develops.

### 1.2. Anaemia in Other Chronic Diseases

Apart from IBD, anaemia is observed in a number of chronic inflammatory disorders. These include autoimmune disorders (e.g., rheumatoid arthritis and celiac disease), cancer and infections. This so-called anaemia of chronic disease (ACD) or anaemia of inflammation is more prevalent in patients with advanced disease and in those responding poorly to therapy [28].

### 1.3. General Health Effects of Anaemia

Treatment of iron-deficiency anaemia in IBD is of importance because of the possible consequences on multiple organs and biological processes. These include cellular dysfunctions comprising impaired mitochondrial respiratory capacity and metabolic impairments that translate into specific organ dysfunctions, for example, in the central nervous system (e.g., impaired cognitive function, fatigue, restless legs syndrome and depression), immune system (e.g., immune cell proliferation and differentiation and regulation of innate and adaptive immune responses), cardiorespiratory system (e.g., reduced exercise capacity, exertional dyspnea, tachycardia, palpitations, cardiac hypertrophy, systolic ejection murmur and risk of cardiac failure), vascular system (e.g., hypothermia and skin pallor), genital tract (e.g., loss of libido and menstrual problems) and gastrointestinal tract (e.g., anorexia, nausea and motility disorders) [32,33]. Collectively, anaemia has a direct impact on the quality of life of affected patients [34,35,36,37].

The perception that anaemia in IBD needs specific treatment apart from regular control of IBD is still underdeveloped. Thus anaemia is neither diagnostically worked up nor do the majority of anaemic patients receive any specific treatment [17], although such an approach is strongly recommended by expert boards and clinical societies [25,38,39]. At present, a great many physicians are uncertain about applicable diagnostic procedures and treatment regimens for their patients with IBD and iron-deficiency anaemia [40]. The aims of this paper therefore are to explore the latest knowledge concerning the pathophysiology of anaemia, diagnostic evaluations, available iron replacement methods and evidence of clinical efficacy in order to provide updated recommendations for the management of iron-deficiency anaemia in IBD. 

## 2. Pathophysiology of Anaemia in IBD

Iron constitutes a key part of haemoglobin in erythrocytes and of myoglobin in muscles, which in combination contain approximately two-thirds of the total body iron. In addition, iron is crucial to a wide range of biological processes [41,42]. The average adult harbours more than 3–4 g of iron, which is balanced between physiologic iron loss and dietary uptake. About 20–25 mg of iron is needed daily for the synthesis of heme. Thus approximately 1–2 mg originates from dietary intake and the remainder is acquired by recycling of iron from senescent erythrocytes by macrophages [41,43]. The total loss of iron averages 1–2 mg/day, mostly through desquamation of intestinal enterocytes or skin, whereas much higher amounts are lost during menstruation [44,45].

### 2.1. Structure of Iron

Dietary iron is available in two forms: heme and non-heme-bound iron. Within heme, iron is complexed as ferrous iron (Fe^2+^) to the protoporphyrin ring, which is abundant in animal food products such as meat, poultry and seafood [46]. Most dietary iron is abundant as nonheme iron (Fe^3+^ or ferric iron) and is present in foods of vegetable origin (e.g., nuts, beans, vegetables and fortified grain products). Heme iron is assumed to constitute 10–15% of total iron intake in meat-eating populations but because of its higher bioavailability (estimated absorption rate of 15–35%) than nonheme iron (5–15%), it accounts for more than 30% of the total absorbed iron [47].

### 2.2. Iron Homeostasis

**Figure 1:**
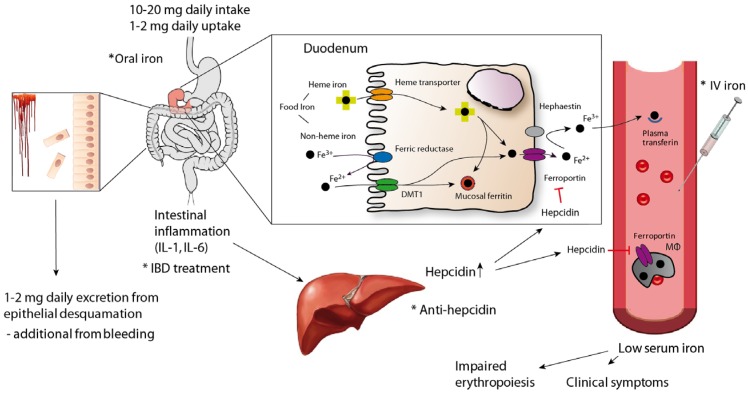
Pathogenesis of iron-deficiency anaemia and methods for supplementation and treatment in inflammatory bowel disease (IBD). IL: interleukin; DMT1: divalent metal-ion transporter 1; MΦ: macrophage; IV: intravenous.

Body iron homeostasis is regulated systemically by several mechanisms, among which is the pivotal interaction of the liver-derived peptide hormone hepcidin with the major cellular iron exporter ferroportin [48]. Ferroportin is found primarily on intestinal epithelium (mostly in the duodenum), macrophages and hepatocytes, which constitute the major cellular iron stores. Ferroportin thus enables the transport of iron from cells into the circulation to maintain adequate systemic iron levels (Figure 1) [49]. Targeting of ferroportin by hepcidin results in ferroportin internalization, degradation and blockage of cellular iron egress into the serum, thus resulting in a reduced availability of iron for erythroid progenitor cells [49]. Synthesis and release of hepcidin and therefore cellular accumulation of iron and development of a low serum iron concentration, are induced by both a high concentration of iron in the liver and plasma and by inflammatory cytokines such as IL-1 (IL: interleukin) and IL-6 [50]. In contrast, during states of iron deficiency, hypoxia and anaemia, the synthesis of hepcidin is blocked in order to increase serum iron levels [29,49,51]. Of note, sexual hormones, alcohol, hepatic function and hypoxia-derived factor all affect hepcidin expression and thus the circulating iron levels [41,42,43]. The efficacy of orally administered iron therapy depends on circulating hepcidin levels. Thus, high hepcidin concentrations may predict nonresponsiveness to oral iron therapy [52]. Hepcidin levels may also control the response to intravenous iron administration, including high-molecular-weight preparations, which are taken up by macrophages and then delivered to the circulation via ferroportin-mediated iron export (Figure 1) [2,21,53]. This is in line with experimental data demonstrating reduced ferroportin expression in the duodenum and decreased iron absorption in individuals with increased hepcidin levels—primarily as a consequence of inflammation [21]. The development of inflammatory anaemia is thus characterized by low circulating iron levels and an iron-restricted erythropoiesis in the presence of high iron stores in the reticuloendothelial system, reflected by normal or elevated levels of ferritin.

Between 1 and 2 mg of iron is taken up daily from the diet, which is balanced by its secretion mainly through intestinal and skin epithelial desquamation. Intestinal bleeding in patients with IBD increases iron loss. With the majority of iron being taken up in the duodenum through heme and nonheme iron transporters (elementary iron is reduced to Fe^2+^ before uptake) with animal data showing some absorption from the large bowel as well [54], the iron enters the epithelial cells. Iron might be stored in the cells as mucosal ferritin or is exported to the circulation through the transporter, ferroportin and oxidized to Fe^3+^. Circulating iron forms complexes with transferrin and is delivered as transferrin-bound iron to cells and tissues. Most of the iron needed for metabolic purposes and erythropoiesis (approximately 20–30 mg/day) originates from macrophages that engulf senescent erythrocytes and reuse iron that is returned to the circulation via ferroportin. During systemic inflammation and increased levels of inflammation-induced hepatic hepcidin secretion, the iron transporter ferroportin found in cells of the reticuloendothelial system and in enterocytes is degraded and cellular iron export is reduced. This results in iron retention in cells of the reticuloendothelial system and impaired dietary iron absorption, subsequently resulting in low serum iron levels with all the clinical consequences mentioned in the text. To overcome iron deficiency in patients with IBD, iron supplementation in the form of oral or intravenous iron can be applied. Novel approaches include inhibition of hepcidin itself or its expression. The most important preventive intervention for long-term well-being of the patients, however, is to efficiently treat the underlying condition, in this case the intestinal inflammatory process. The asterisk indicates points of therapeutic intervention. 

### 2.3. Inflammatory Modulators in Anaemia

Cytokine-driven induction of hepcidin expression and the direct effects of cytokines on iron trafficking in macrophages and duodenal enterocytes play a decisive role in the development of ACD) or anaemia of inflammation by retaining iron in the reticuloendothelial system and blocking iron absorption, causing an iron-limited erythropoiesis [28,55]. Thus, ACD is more prevalent in patients with advanced disease and in those responding poorly to therapy [28]. In addition, cytokines and chemokines further contribute to anaemia by negatively influencing the biological activity of erythropoietin, by inhibiting the proliferation and differentiation of erythroid progenitor cells and by reducing the circulatory half-life of erythrocytes [28].

However, patients with flaring IBD experience chronic blood loss due to intestinal mucosal bleeding, which often causes true iron deficiency in conjunction with inflammatory anaemia (mirrored by low to normal ferritin levels) [22,56]. Of note, while hepcidin levels are increased in patients with ACD, concomitant true iron deficiency results in hepcidin suppression [21]. On the one hand, this is due to the fact that iron deficiency inhibits SMAD-mediated signalling pathways in hepatocytes, thereby blocking hepcidin expression even in the presence of inflammatory stimuli such as IL-6 [57]. On the other hand, anaemia and hypoxia result in activation of hormones that have a negative impact on hepcidin formation. These include erythroferrone produced by erythroblasts in response to erythropoeitic stress [58], as well as other mechanisms, including growth differentiation factor 15 (GDF-15), which is seen mainly in patients with hemoglobinopathies. Further, a hypoxia-driven blockade of hepcidin formation is induced via platelet-derived growth factor-BB (PDGF-BB) and/or hypoxia-inducible factors (HIFs) [58,59,60,61,62]. Together these mechanisms result in increased circulating iron levels via stimulation of iron absorption and redelivery from macrophages. Thus, in the presence of both inflammation and true iron deficiency due to intestinal bleeding in IBD, circulating hepcidin levels decrease because anaemia and iron-deficiency regulatory signals dominate over inflammation-driven hepcidin induction [62,63]. Therefore, truly iron-deficient patients, even in the presence of systemic inflammation, are able to absorb considerable amounts of iron from the intestine [21,49].

Finally, vitamin deficiencies (e.g., vitamin B_12_, folic acid and vitamin D) due to either intestinal inflammation or extensive bowel resection can also contribute to the development of anaemia [22,64], as do specific medications for the treatment of IBD (as listed earlier).

## 3. Diagnostic Investigations

According to the World Health Organization (WHO), adult males and females with a blood haemoglobin concentration below 13 and 12 g/dL, respectively, are considered anaemic (<11 g/dL during pregnancy) [31]. Thresholds for defining the state of anaemia apart from sex and pregnancy, however, depend on such factors as age, altitude and ethnicity. The diagnosis of iron deficiency and anaemia is based on measurements of the blood haemoglobin concentration but some additional basic analyses are required for a diagnostic workup and for tailoring optimal therapy in patients with IBD [29,38,65]. During flaring IBD, measurements of iron status may be difficult to interpret because parameters relating to iron metabolism are influenced by the inflammation per se [66].

### 3.1. Transferrin and Transferrin Saturation

As a consequence of chronic inflammation, patients with active IBD may show reduced levels of transferrin, which is contrary to the definition of patients with iron deficiency [67]. Importantly, patients with inflammatory anaemia with or without true iron deficiency are characterized by reduced serum iron and a low transferrin saturation (TfS) (i.e., the quotient of iron concentration (µmol/L) divided by transferrin concentration (mg/dL) in fasting blood samples multiplied by 70.9 and stated as a percentage) [68]. Accordingly, a number of studies have used TfS as an indicator for low iron status and for determining appropriate initiation of iron supplementation therapy [37,38,69,70,71]. A TfS of 16% is generally used as a threshold when screening for iron deficiency, although a 20% threshold is often applied in the context of coexisting inflammatory disorders [65].

### 3.2. Ferritin

Serum ferritin concentration, which generally correlates with body iron stores, is the most widely used surrogate marker of stored iron and works nicely in patients without any concomitant inflammatory condition. Circulating ferritin levels, however, are influenced by inflammation; in fact, several proinflammatory cytokines stimulate ferritin expression, leading to measurements in the normal or even elevated range during chronic inflammation, even in the presence of true iron deficiency [66,72,73]. Thus, in situations of concomitant inflammation, chronic liver disease, or malignancy, ferritin levels may increase independently of iron status. Thus, independent of inflammation, a ferritin concentration below 30 µg/L is indicative of true iron deficiency [74,75], whereas this threshold may be higher in patients with inflammation, although prospective and interventional studies examining this issue in detail are lacking. In clinical practice, a ferritin level of up to 100 µg/L in the setting of an inflammatory disease and anaemia may be associated with true iron deficiency, whereas functional iron deficiency may be present with ferritin levels exceeding 100 µg/L in such conditions [68]. Currently, no standard clinical tests exist for the assessment of true iron deficiency in patients with concomitant inflammation and thus a combination of various paraclinical tests is often required to provide clinical evidence of iron deficiency and to guide therapy [68,76].

### 3.3. Soluble Transferrin Receptor

Serum-soluble transferrin receptor (sTfR), a proteolytic derivative of the membrane-bound transferrin receptor, is another marker of iron status. With true iron deficiency, increased synthesis of transferrin receptors is observed along with a corresponding increase in sTfR levels. Nevertheless, the sTfR concentration might also rise during disorders associated with increased erythropoiesis, including chronic lymphatic leukaemia, whereas it can be reduced by the actions of cytokines during inflammation. Therefore, no consensus currently exists as to a standardized cut-off value for sTfR [77]. To distinguish between patients with inflammation-driven ACD and patients with ACD and concomitant true iron deficiency, determination of the ratio between sTfR and the logarithm of serum ferritin concentration (i.e., the sTfR-F index) has been recommended [78]. A sTfR-F index above two is indicative of true iron deficiency among ACD patients, whereas a ratio below one is suggestive of ACD alone without concomitant iron deficiency [68,77].

### 3.4. Red Cell Indices

Based on erythrocyte analyses of full blood samples from anaemic patients, information about mean cellular haemoglobin concentration (MCHC, hypochromia) and mean cell volume (MCV, microcytosis) can be obtained. The values of these indices are decreased by iron deficiency. In the case of microcytosis in patients with an appropriate ethnic background, haemoglobin electrophoresis may be considered to rule out hemoglobinopathies such as sickle-cell disease and thalassemia [77]. Microcytosis or MCHC reductions thus may indicate true iron deficiency in patients with inflammation-associated ACD because classical ACD is characterized as normochromic and normocytic [66,67,70,76]. Among patients with chronic kidney disease, measuring the percentage of circulating hypochromic red cells as a proportion of total red blood cells can indicate the presence of iron deficiency using a cut-off value of 6% [79]. Unfortunately, freshly drawn blood samples and specific equipment are required for this analysis [80]. Accordingly, prospective evaluations of this parameter, as well as of the reticulocyte haemoglobin content in clinical situations, including patients with anaemia and inflammatory disorders, are scarce [68,81].

### 3.5. Bone Marrow Analyses

Bone marrow aspiration for diagnosing iron deficiency appears to be the gold standard. This method is thought to be unaffected by inflammation but it is invasive in nature, uncomfortable for the patient, expensive and might be affected by concomitant treatment with recombinant erythropoietin. Thus, bone marrow aspiration should be reserved for specific situations where other techniques are either unavailable or conflicting [77].

### 3.6. Hepcidin

Hepcidin has a key regulatory role in iron homeostasis as an inhibitor of cellular iron export [77]. Measurements of hepcidin may be an attractive tool to diagnose true iron deficiency in patients with inflammation-driven anaemia because its expression in vivo appears to be more affected by iron deficiency than by the inflammatory response [62,82]. Recently, commercially available tests have been introduced into clinical practice [83] but the usefulness of hepcidin determination to correctly indicate iron deficiency in patients with inflammation needs to be tested prospectively in future studies. Of note, concomitant pathologies that may contribute to the development and severity of anaemia, including folic acid, cobalamin, or vitamin D deficiency and haemolysis and erythropoietin deficiency per se, as well as any renal insufficiency, must be identified and treated properly, if possible.

It is of great importance to establish the presence of true iron-deficiency anaemia in patients to avoid any unnecessary treatment. The assessment should be guided by predictive serum parameters such as hepcidin, soluble transferrin receptor and others including red cell indices but no gold standard is available at present [76,84]. The success of treatment with either oral or intravenous iron is mirrored by an increase of haemoglobin levels or by increased levels of circulating ferritin.

## 4. Treatment of Anaemia

The primary treatment of ACD is to cure the underlying pathology or other easily treatable conditions contributing to anaemia, such as vitamin deficiency, which often leads to improvement in haemoglobin levels unless other pathophysiologic factors or deficiencies are present [14,25,28,29,33,85]. In cases of severe anaemia (i.e., haemoglobin < 7–8 g/dL) [2,86], especially when it is rapidly developing, as in association with acute gastrointestinal bleeding, or if the patient suffers from comorbidities such as coronary heart disease or chronic pulmonary disease, a rapid correction of haemoglobin levels may be indicated, which can best be achieved with red blood cell transfusions [25,28,33,87]. However, the use of blood transfusions must be considered carefully because negative effects are well documented [87,88] and a liberal application of transfusions is associated with higher mortality in patients with acute gastrointestinal bleeding [89]. Moreover, transfusions have been associated with an increased risk for nosocomial infections and mortality rates among intensive care patients [90], as well as a higher frequency of surgical-site infections [91]. Additionally, a risk of transfusion-related anaphylactic reaction, together with a small but residual risk for transmitting infectious disease, does exist [92,93,94].

## 5. Iron Replacement Formulations 

Imbalances of iron homeostasis are the major reason for anaemia in patients with IBD. The currently available options for iron supplementation to balance iron intake and iron loss consist of oral and intravenous administration and their pros and cons are listed in Table 1.

### 5.1. Oral Regimen

**Figure 2:**
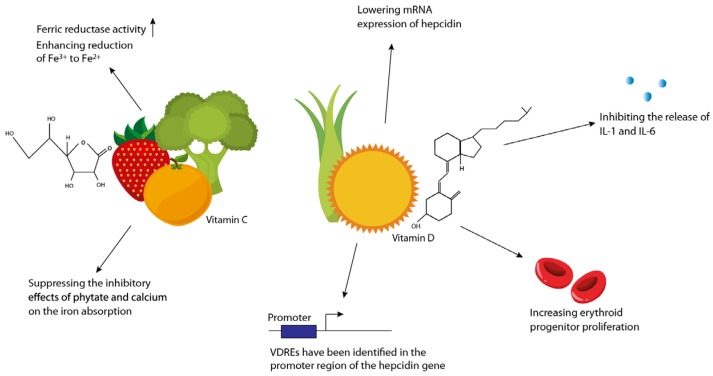
Importance of vitamins C and D in the treatment of iron-deficiency anaemia.

The bioavailability of “traditional” oral iron preparations is relatively low but nevertheless is the first-line therapy in iron-deficiency anaemia. Oral iron has a well-established safety profile, is easy to administer and comes with a generally low cost—with the latter being important in a pharmaco-economical setting [26]. Oral iron supplements are available as divalent Fe^2+^ (ferrous) or trivalent Fe^3+^ (ferric) salts coupled with sugar complexes or protein succinylate [95,96]. The most widely used preparations are ferrous sulphate, ferrous gluconate and ferrous fumarate, which all contain the ferrous form of iron, which has a better bioavailability than ferric-containing formulations [96]. Prior to absorption by enterocytes in the duodenum, iron is reduced to its ferrous state (Fe^2+^)—A process catalysed by membrane-bound ferric reductase. Divalent metal transporter-1 (DMT1) facilitates iron uptake in the acidic environment [97]. Ascorbic acid (or vitamin C) dose dependently facilitates absorption of oral iron [98] by providing reducing equivalents for ferric reductase, thus enhancing the reduction of Fe^3+^ to Fe^2+^ prior to epithelial uptake [99]. Further, vitamin C suppresses the negative effects on iron absorption of inhibitors such as phytate and calcium [47] (Figure 2). 

Recently, a study in children has shown that supplementation of vitamin D facilitates increased haemoglobin levels. Here plasma concentrations of 25-hydroxyvitamin D (25(OH)D) below 30 ng/mL (i.e., vitamin D deficiency) were associated with increased hepcidin concentrations and reduced haemoglobin concentrations compared with individuals with plasma 25(OH)D concentrations above 30 ng/mL [100]. Because vitamin D deficiency is frequent in IBD [101] and because vitamin D has been shown to inhibit hepcidin expression [102] and to possess important immunologic effects of benefit in the clinical course of patients with IBD [103,104,105], normalization of vitamin D is important for elevating the haemoglobin level in these patients. Specifically, vitamin D binds to vitamin D response elements (VDREs) in the promoter region of hepcidin (hepcidin antimicrobial peptide, HAMP) and thereby reduces hepcidin expression [102,106,107,108]. Supplementing healthy adults with vitamin D decreased hepcidin levels by 73% [109] and significantly increased haemoglobin levels in critically ill patients following administration of up to 100,000 IU of vitamin D daily for 5 days [107]. These recent results highlight the importance of vitamin D in the context of anaemia (Figure 2).

Dietary vitamin C enhances iron absorption by providing reducing equivalents for Fe^3+^ reduction by the enzyme ferric reductase to enhance its activity. Vitamin C also suppresses the inhibitory features of phytate and calcium on iron uptake. Vitamin D obtained from the diet or generated in the skin through ultraviolet-induced photolysis of vitamin D precursors augments iron absorption by lowering mRNA expression of hepcidin mediated by the presence of vitamin D response elements (VDREs) identified in the promoter region of the hepcidin gene. Additionally, vitamin D inhibits the release of IL-1 and IL-6 and increases erythroid progenitor proliferation.

Recently, new orally available products have been introduced into clinical practice. One of these, ferric maltol, has been successfully studied in a phase III trial in IBD patients with iron-deficiency anaemia who had previously been either intolerant or unresponsive to oral ferrous products [110].

Although the optimal dose of oral iron supplements in patients with IBD and iron deficiency has not been established, a dose of 50–200 mg/day of elemental iron is often recommended [111]. Only 10–25% of the dosed iron is expected to be absorbed in iron-deficient patients [71,97]. Because oral iron induces the expression of hepcidin, it appears reasonable to dose oral iron only once daily to circumvent the inhibitory effects of hepcidin on iron transfer from duodenal enterocytes to the circulation. A recent observational trial confirmed this notion. It was shown that oral administration of iron reduced the level of iron absorption on the following day and that application of iron twice daily resulted in a significant reduction in oral iron bioavailability. Of note, the relative percentage of absorbed iron could be increased even by administration every second day [112]. This observation, combined with a very recent study in women with depleted iron stores demonstrating that alternate-day administration of oral iron supplementation resulted in higher fractional as well as total iron absorption compared with daily administration [113], may alter the current practice of oral iron administration.

Given the low absorption of oral iron, a high proportion remains in the gut and is associated with the development of gastrointestinal side effects, including nausea, dyspepsia, diarrhoea, abdominal discomfort, vomiting and constipation in up to 20% of patients—often resulting in nonadherence to therapy [26,114]. Generally, nausea and abdominal discomfort occur within 1–2 h of drug intake and appear to be dose related, whereas other gastrointestinal side effects such as diarrhoea and constipation are idiosyncratic [71,111]. In patients reporting such intolerances, a delayed-release enteric-coated iron tablet may be prescribed. However, the bioavailability of iron from these formulations is reduced compared with standard preparations because almost all the iron is absorbed in the duodenum and not in distal part of the gastrointestinal tract [45,47]. Moreover, because oral iron is poorly absorbed in the setting of ongoing inflammation [21], IBD patients with increased C-reactive protein (CRP) levels often show a diminished response to oral iron therapy [115].

Most of the reservations regarding oral iron therapy in IBD come from studies in animal models, which have shown contradictory evidence regarding the impact of oral iron on ongoing intestinal inflammation [35,116]. In humans, the clinical evidence for the effects of oral iron in patients with flaring IBD is also controversial [38,117]. It is, however, established that iron therapy significantly affects the composition of the microbiome [118,119]. This is of interest because the composition of the microbiome is regarded as an important factor in the pathogenesis of IBD [120]. A recent open-label clinical trial showed a significant impact of iron supplementation (both oral and intravenous) on the phylogenetic composition and faecal metabolite landscape in patients with IBD and iron deficiency or anaemia [118]. A difference between the impact of orally and intravenously administered iron on bacterial phylotypes and faecal metabolites seems to exist and this may relate to differences in iron pharmacokinetics and iron availability for gut bacteria. Moreover, patients with CD appear to be more prone to changes in microbiome composition following iron replacement therapy and intravenous iron therapy might in fact benefit such anaemic patients with an unstable microbiota [118]. Based on these findings, iron ingestion may potentially influence the disease course in patients with IBD [118,121].

### 5.2. Intravenous Regimen

Parenteral iron administration more rapidly increases haemoglobin levels than oral delivery [122,123] and this option traditionally has been reserved for patients who are intolerant to or respond inadequately to oral iron supplementation, as well as for patients in whom a rapid iron replenishment is desired (e.g., patients scheduled for surgery) [25,85,114]. This approach is reflected by the indications approved by the U.S. Food and Drug Administration (FDA) and the European Medicines Agency (EMA) for a number of intravenous iron preparations [124,125]. In the past, when high-molecular-weight dextrans were used for intravenous iron therapy, infrequent severe or life-threatening anaphylactic reactions were reported following intravenous administration [126]. However, the risk of these severe adverse events is lower today with the currently used preparations including high-molecular-weight iron components [127]. Compared with oral administration, intravenous iron increases haemoglobin levels and iron storage and improves quality of life more rapidly but not always more effectively [119,128,129]. The disadvantages—apart from a higher cost of therapy—include a risk of infusion-related anaphylaxis, which means that equipment to manage such potentially life-threatening situations must be in place [127]. Moreover, intravenous iron has been recommended in favour of oral iron therapy in patients with more advanced inflammation to bypass the blockade of iron absorption by hepcidin, although clinical data are scarce in proof of concept of this suggestion. Patients with more advanced inflammation/severe disease activity have often been excluded from prospective clinical trials evaluating the efficacy of intravenous iron in IBD. Of note, a retrospective analysis of results from various clinical trials suggested that pre-treatment CRP levels are not significantly associated with therapeutic responses to intravenous iron [115].

Six intravenous iron preparations are available at present. These include iron dextran, iron gluconate and iron sucrose, as well as the more recently licensed high-molecular-weight compounds ferumoxytol, iron isomaltoside 1000 and ferric carboxymaltose [26,130,131]. The structural stability of these high-dose preparations is high and allows only the release of a low level of labile iron into the circulation, resulting in improved safety profiles and infusion of higher iron dosages. In most patients, the total iron dose required therefore can be provided in a single infusion.

#### 5.2.1. Low-Molecular-Weight Iron

The iron dextran compounds exist in two forms that are stable: A low- (73 kDa) and a high-molecular-weight (165 kDa) complex. Because the latter has been linked to an increased risk of both anaphylactic and anaphylactoid reactions [132,133,134,135], only the low-molecular-weight iron dextran is currently marketed in Europe [136]. This form can be administered as a single dose of up to 200 mg over a minimum infusion period of 30 min [137]. Previously it was recommended first to administer a test dose to check for the risk of anaphylactic reactions (i.e., 0.5 mL over 2–5 min) before providing the full dose but this precaution is no longer recommended by the EMA [124].

The stability of both iron gluconate (37 kDa) and iron sucrose (43 kDa) is lower than that of iron dextran and these two iron compounds can be administrated at a maximal single dose of 200 mg of iron gluconate (300 mg in some countries) over a minimum infusion time of 30 min [138] or 62.5 mg of iron sucrose (125 mg in some countries) over an infusion time of 5–10 min [139] without requiring a test dose. Increasing the dosages [140] or the infusion rates [53] enhances the risk of adverse events such as transient hypotension due to the release of labile iron. Accordingly, iron dextran, iron sucrose and iron gluconate preparations usually will require multiple rounds of administration with lower doses to replenish iron stores.

#### 5.2.2. High-Molecular-Weight Iron Compounds

The introduction of more stable iron complex formulations for intravenous iron administration has permitted the infusion of higher single doses with minimal side effects and no need for test doses because of the marginal release of labile iron during administration. The highly stable 150 kDa complexes ferric carboxymaltose [141,142,143,144,145] and iron isomaltoside 1000 [146,147] allow for controlled and safe delivery of higher doses of molecular iron per infusion. Ferric carboxymaltose may be administered effectively at a dose up to 1000 mg over a period of at least 15 min once per week [146]. Iron isomaltoside 1000, because of its stable structure, can be administrated in single doses of up to 20 mg/kg of body weight within a period of 15 min [146]. Currently, limited data exist on iron isomaltoside 1000 for the treatment of iron-deficiency anaemia in patients with IBD [129,147], although clinical trials are currently ongoing. Ferumoxytol has a molecular weight of 721 kDa, which allows for rapid dosing of relatively large doses [148]. A recent phase III randomized, double-blind, placebo-controlled trial conducted at 182 sites in the United States, India and Europe evaluated administration of 510 mg doses of ferumoxytol followed by a second dose 2–8 days later in 231 patients with various gastrointestinal conditions (including IBD, polyps and colon cancer). In this study, ferumoxytol was efficacious and generally well tolerated in patients with iron-deficiency anaemia along with underlying gastrointestinal disorders who had a history of unsatisfactory oral iron supplementation [149]. However, with respect to IBD, it has been suggested that the paramagnetic nature of ferumoxytol might lead to interference during magnetic resonance imaging (MRI) examinations [150] and such interference might hamper its use in a subset of IBD patients because MRI examinations are an important diagnostic tool in their management. Further, a comparison of different intravenous iron products in the United States showed that ferumoxytol, per sold unit, had the highest rate of adverse events [151], impeding its benefit-risk ratio. In addition, since March 2015, a boxed warning by the FDA has been attached to this product regarding potentially life-threatening allergic reactions.

Although different side-effect profiles are associated with various preparations of large-molecule iron complexes [133], the most frequently reported complaints after infusion are itching, dyspnea, wheezing and myalgia [152]. Moreover, it should be noted that acute myalgia following a first intravenous iron administration (without any other symptoms) ceases spontaneously within minutes (i.e., the so-called Fishbane reaction) and does not recur at re-challenge [152,153]. In addition, more specific side effects include hypotension, tachycardia, dyspepsia, diarrhoea, stridor, nausea, skin flushing and periorbital oedema. Serious side effects are rare following intravenous iron infusion [154] but can include cardiac arrest [155]. The risk is increased among elderly patients and has been observed most often following infusion of high-molecular-weight dextran-containing preparations that are no longer in clinical use [156]. Accordingly, an initial low infusion rate is advisable, as well as a close monitoring of patients for signs of hypersensitivity both during administration of an intravenous iron formulation and for at least 30 min thereafter [124]. 

Based on our expanding knowledge of the pathways underlying inflammatory anaemia and specifically the role of hepcidin, new therapeutic strategies are emerging that attempt to block hepcidin activity either by directly interfering with hepcidin synthesis by affecting different inflammation- or iron-driven signalling pathways that regulate hepcidin expression (such as SMAD, STAT3, BMP, BMPR, or TMPRSS6) or by neutralizing hepcidin in the circulation [57,157,158,159,160] (Figure 1). Such interventions are currently under clinical investigation but they can only be effective in patients with inflammation- or renal insufficiency- driven hepcidin elevation and subsequent iron retention in macrophages, where hepcidin antagonization will result in redistribution of iron to the circulation and delivery of the metal to erythroid progenitors. In patients with true iron deficiency in the setting of inflammation, which is often the case in IBD, such therapies will not work and iron supplementation will remain the treatment of choice. Another set of new drugs has arisen from the development of prolyl-hydroxylase inhibitors. These therapeutic agents cause stabilization of HIFs, resulting in increased endogenous erythropoietin formation and stimulation of iron uptake based on the regulatory effects of HIFs on the expression of transmembrane iron transporters. These agents are currently being investigated in clinical trials mainly to combat renal anaemia [161,162,163].

## 6. Evidence of Management

Members of our group previously performed a systematic search that yielded a total of 632 studies concerning iron therapy in IBD published from January 2004 to March 2015 (i.e., in a time frame with novel high-dose intravenous iron preparations), of which 13 prospective trials met the inclusion criteria as randomized, controlled trials and included 2906 patients in total [164]. This systematic review indicated that administration of intravenous iron in IBD patients with mild anaemia (haemoglobin ≥ 10 g/dL) frequently resulted in higher ferritin levels but not in higher haemoglobin concentrations compared with oral iron supplementation at short-term follow-up [129,144,145,165]. In more aggravated iron-deficiency anaemia, intravenous iron supplementation was superior to oral treatment when the evaluation was based on the increase in haemoglobin [128,134,145,165].

Comparative studies of intravenous versus oral iron supplementation in the systematic review did not demonstrate any significant difference in haemoglobin normalization favouring the use of intravenous iron therapy unless considered for patients with intolerance or an inadequate response to oral supplementation [164]. In patients undergoing biological therapy with TNF inhibitors, concomitant iron supplementation may be prescribed without affecting the disease course/activity. Moreover, another recent systematic review of randomized, controlled trials with the aim of assessing drug safety demonstrated that intravenous iron therapy may increase the risk of infection [166]. This issue has also been evaluated in predialysis and dialysis patients indicating differences in the risk of infection based on baseline ferritin levels, mode of administration (intermittent or bolus) and the specific drugs used [167,168,169,170,171].

It is known that apart from the WHO definitions of anaemia [31], a low TfS in fasting blood samples (<20%) and a serum ferritin concentration of less than 30 µg/L (with a serum CRP level within the normal range or a ferritin concentration of less than 100 µg/L with an elevated serum CRP level) are suitable laboratory tests for the diagnosis and assessment of iron deficiency in IBD used in the randomized, controlled studies [164].

Only nine randomized, controlled trials investigating oral iron supplementation in IBD patients were published between 2004 and 2017 [35,110,128,129,134,138,144,145,165]. Oral supplementation appears to be well tolerated and has a positive effect on both haemoglobin levels and body iron parameters. From these studies, it seems that milder side effects (i.e., abdominal discomfort, diarrhoea, nausea and vomiting) occur less often after intravenous therapy than after oral therapy [128,134,138,144,165], although one study did not report any differences [129]. No comparison of side effects based on the various forms of oral supplementation was, however, performed. From an examination of the available data, it was apparent that there are no indications that oral iron supplementation exacerbates symptoms of the underlying IBD. Only one study in this systematic review [164] reported worsening of disease activity in 2 of 33 patients with UC (but not in patients with CD). However, in this study, the IBD quality-of-life scores improved significantly (*p* = 0.016) at the same time [35] and when the eight studies using oral iron supplementation were evaluated, it was apparent that an adequate level of evidence is provided to verify the safety of oral iron supplementation in IBD. Of note, a study with oral ferric maltol has suggested that this drug may be an alternative for patients who are unresponsive to or intolerant of formulations containing ferrous salts [110], an observation that needs to be confirmed in future studies, though.

A very recent systematic review and Bayesian network meta-analysis performed on the five eligible randomized, controlled trials with a total population of 1143 patients has shown ferric carboxymaltose to be the most effective preparation for the treatment of iron-deficiency anaemia in IBD, followed by iron sucrose, iron isomaltoside and oral iron in fourth place [172]. This analysis incorporated all currently available evidence on intravenous iron replacements in IBD patients with iron-deficiency anaemia and is the first attempt to systematically and quantitatively review the literature in the field.

It is generally accepted that individuals with iron deficiency and coexisting anaemia need treatment. It is, however, a subject of debate whether treatment of iron deficiency should be initiated before the development of anaemia—a condition that recently was reported to occur in 37% of IBD patients in a Spanish outpatient cohort [173]—because data from clinical trials on this issue are scarce. Thus, a placebo-controlled, double-blinded, randomized study in women with iron deficiency but without anaemia indicated that intravenous iron administration resulted in an improvement of fatigue in 82% of patients in the intervention group compared with 47% in the placebo group and that the effect of iron supplementation on fatigue was most pronounced in women with an initial ferritin concentration of less than 15 ng/mL [174]. Similar beneficial effects of intravenous iron administration regarding quality of life in non-anaemic patients with IBD have recently been published [175,176]. Thus, none of these observational, single centre studies included a placebo control given the high incidence of placebo mediated benefits on quality of life in such patient cohorts [174,177]. Nevertheless, it has to be kept in mind that uncritical iron supplementation or iron overloading may have several adverse effects described herein, including allergic reactions, risk of infections or intravascular oxidative stress as well as impairment of mitochondrial function with subsequent fatigue [178]. This leads to the yet unsolved question of therapeutic start and end points in terms of target haemoglobin and/or sTfS/ferritin levels and whether or not full correction of anaemia is optimal for patients with inflammation-associated anaemia. Nevertheless, patients with concomitant diseases such as congestive heart failure and fatigue due to true iron deficiency may benefit from such iron supplementation [70].

## 7. Recommendations for Clinicians

The cause of anaemia and specifically of concomitant iron deficiency should be identified in every patient with IBD. Thus, the recurrence of iron deficiency following successful treatment is often due to persistence or relapse of the initial inciting cause (e.g., recurrent gastrointestinal or urogenital bleeding), which should be managed appropriately. Further, in patients who have failed to respond to either oral or parenteral iron therapy, the cause for this failure should be carefully determined.

Previously it was accepted that clinical symptoms of anaemia occurred only when the haemoglobin level dropped abruptly [37] and, conversely, that patients would adapt to low haemoglobin levels if the anaemia developed slowly. This led to the concept of asymptomatic anaemia. In truth, the term asymptomatic seems to reflect the fact that impairments in physical condition, quality of life, cardiovascular performance and cognitive function may have been neglected by both patients and their physicians. Therefore, the process of adaptation in chronic anaemia seems to be an acceptance/toleration of impaired quality of life [37] and chronic fatigue and reduced physical activity/cardiovascular performance caused by anaemia may actually debilitate and even worry patients with IBD as much as abdominal pain or diarrhoea [37]. Accordingly, the beneficial effect on quality of life and metabolic processes derived from the correction of anaemia in patients with IBD may be just as important as the control of their intestinal disease [37].

### 7.1. Oral versus Intravenous Iron Supplementation

Clinical guidelines often emphasize that because of the ease of treatment, patients with uncomplicated iron-deficiency anaemia should be treated with oral rather than intravenous iron formulations [179]. In this context, an appropriate dosage for treatment of iron deficiency in adults is usually recommended in the range of 100–200 mg/day of elemental iron but guidelines do not consider that a number of side effects are dose related and might be prevented by reducing the dosage to as low as 50 mg of elemental iron per day in selected patients, which, in fact, may be sufficient to correct mild iron-deficiency anaemia [180].

Indications for intravenous iron administration include severe anaemia (haemoglobin < 10 g/dL), intolerance of or inappropriate response to oral iron administration, severe intestinal disease activity and concomitant therapy with an erythropoiesis agent or patient preference. Oral iron supplements can be used if these indications for intravenous therapy are not met.

If intravenous iron supplementation is considered, the use of low-dose regimens is not recommended from the point of view of clinical efficacy because a number of infusions might be needed over several days or weeks. Instead, high-dose regimens that result in fewer infusions and increase both convenience and cost-effectiveness of intravenous iron repletion should be considered.

The optimal dosing strategy for intravenous iron compounds depends on the type of preparation, the body weight of the patient and the haemoglobin concentration. The amount of iron needed to correct the haemoglobin can be calculated using the Ganzoni equation [181], often regarded as the gold standard, although this formula might underestimate the iron needed when a target haemoglobin of 13 g/dL and stored iron of 500 mg are used to determine individual iron deficits [129]. Because this formula is inconvenient in clinical practice [129,144], simpler schemes for the estimation of total iron need have been published [38,182], including a simple regimen to predict individual iron requirements for ferric carboxymaltose [142] that may also be used in clinical practice for dosing of other intravenous iron preparations [38]. It should be mentioned that patients with iron-deficiency anaemia who are unresponsive even to intravenous iron supplementation (i.e., haemoglobin increase ≤ 2 g/dL within 4 weeks) may in addition need recombinant erythropoiesis-stimulating agents after ruling out other causes of anaemia such as vitamin deficiencies [183,184,185].

### 7.2. Surveillance of Patients Following Iron Supplementation

Last but not least, it should be kept in mind that iron deficiency in IBD often relapses after iron replenishment [143]. Consequently, periodic monitoring, for example, every 3 months during treatment and again after a year once the haemoglobin value is normalized and iron stores are replenished (i.e., preventive treatment), is essential to assess whether retreatment is required [73]. Such a proactive concept of anaemia management not only could improve the quality of life for patients with IBD but also could be of economic benefit. However, we lack solid data on when to stop iron supplementation therapy in order to avoid iron overloading, which may cause side effects due to iron-catalysed formation of toxic radicals [31]. Recent guidelines on the management of anaemia among dialysis patients suggest that ferritin levels of up to 500 ng/mL appear to be safe and this also might be a useful upper threshold in the management of patients with IBD and anaemia [186].

This leads to the questions of (1) therapeutic start and end points (i.e., when should iron supplementation therapy be initiated and when it should be discontinued?) and (2) whether or not iron-deficiency anaemia should be treated differently depending on the underlying disease? To start with the latter point, in general, subjects with pure iron-deficiency anaemia on the basis of an inadequate dietary iron intake and/or increased blood losses and iron-deficient IBD patients with no or minimal signs of inflammation should initially be recommended to oral iron therapy. However, such a recommendation might have some caveats. Oral iron replacement therapy may be of limited efficiency in the setting of concomitant inflammation, which is usually associated with increased hepcidin concentrations resulting in an impaired response to iron therapy [84,187]. Yet true iron deficiency in the setting of inflammation causes hepcidin reduction and enables duodenal iron absorption, although to a lesser extent than in healthy control individuals [21,62,63]. Second, iron supplementation may be a problem in areas with an endemic burden of infectious disease or in patients with active infections because iron is an essential growth factor for many microbes and also has an impact on antimicrobial immune responses [188]. Thus, dietary iron fortification strategies were associated with an increased risk of infections such as malaria, bacterial meningitis, bacterial pneumonia and viral diarrhoea along with a rise in infection-related mortality [189,190].

### 7.3. General Precautions for Iron Supplementation

While normalization of haemoglobin appears to be a reasonable readout in subjects with iron-deficiency anaemia in the absence of inflammation [31], retrospective data, mainly from patients with chronic kidney disease, who are also characterized by a low-grade inflammation, indicate that haemoglobin normalization seems to be associated with an increased mortality compared with subjects with mild anaemia [28,191,192]. This has resulted in recommendations from different societies that in the presence of an inflammatory disease, including cancer or autoimmune disorders, the target haemoglobin concentration should be slightly below normal [28,31]. However, this is an extrapolation of data from observational studies and it is still unknown whether this is also true for patients with IBD.

Importantly, life-threatening reactions possibly caused by release of free iron are rare after administration of intravenous iron supplementation therapies [127,193]. Thus, practical recommendations for minimizing the risk of hypersensitivity reactions, for example, by assessing any previous adverse reactions, multiple drug allergies, or severe atopy, should be applied. Also, decreasing the infusion rate as well as maintaining an appropriately staffed site equipped with resuscitation facilities may be considered [127]. An incompletely understood issue is the development of hypophosphatemia in some patients specifically in association with ferric-carboxymaltose administration. Infrequently, hypophosphatemia may become severe and life threatening and may be linked to alterations of the FWF23 and vitamin D pathways, although the details of that network, as well as measures to identify patients at risk, are not available thus far [194].

Patients with inflammatory diseases will respond poorly to oral iron therapy unless the iron deficiency is severe. Newer iron formulations, such as ferric maltose, have been shown to correct mild anaemia in patients with quiescent IBD [110] but whether this is also true in flaring IBD still remains to be established. Nevertheless, in patients with inflammation and anaemia based on iron-limited erythropoiesis and in patients with non-inflammatory-driven severe iron-deficiency anaemia, in whom a fast recovery of depleted iron stores is desired, intravenous iron appears to be the treatment of choice. Still, the evidence from studies in proof of this latter concept is rather scare [179] and we still lack data from prospective trials on the efficacy of intravenous iron preparations in patients with more advanced inflammation.

## 8. Conclusions

Here we have summarized the impact and pathophysiology of iron deficiency in the setting of IBD. Diagnostic criteria are provided as well as methods to differentiate between functional and true iron deficiency. We also discussed the currently available drugs and commented on issues that should be considered by physicians treating patients with IBD. Thus, treating physicians need to pay more attention to the management of anaemia and iron deficiency for improvement of the general well-being of their patients with IBD—a matter that actually does not gain the attention it deserves. Although we lack knowledge on the effects of iron repletion strategies on the course of IBD, the control of inflammation is pivotal in the management of anaemia in this intestinal disorder.

Given the novel intravenous high-dose iron replacement regimens introduced within the last decade, oral iron therapy should be preferred for IBD patients with mild and uncomplicated iron-deficiency anaemia (haemoglobin ≥ 10 g/dL) in quiescent disease stages unless previous complications have been observed, including an inadequate response (haemoglobin increase < 2 g/dL within 4 weeks) [195]. Intravenous iron supplementation may be of advantage in patients with aggravated iron-deficiency anaemia or flaring IBD (haemoglobin < 10 g/dL) because inflammation hampers intestinal iron absorption [27,196,197]. Further, based on the available data, iron therapy can be administered concomitantly with TNF inhibitors [198], a class of drugs widely used in the management of IBD [199]. When using intravenous iron preparations, physicians must be aware of infusion-related side effects and the risk of hypophosphatemia. Further, efficacy studies of intravenous iron preparations in patients with more advanced inflammation are urgently desired.

Finally, it should be emphasized that iron deficiency may relapse often after iron replenishment [143], specifically when IBD activity is not well controlled and consequently, periodic monitoring should be highlighted to assess whether retreatment is required [73]. However, we still lack solid data on when to stop iron supplementation therapy in order to avoid side effects due to iron overloading. Thus large, well-designed collaborative prospective trials involving scientists and physicians from different disciplines are warranted to assess the true impact on the management of IBD associated with iron-deficiency anaemia.

## Figures and Tables

**Table 1:** Main principles of iron supplementation and their pros and cons.

Iron Administration	Pros	Cons
Oral	Low cost Convenient Available over the counter Efficient when intestinal absorption is not impaired	Mucosal injury Alteration of microbiota Various disorders may impair uptake, e.g., celiac disease, ACD *, autoimmune gastritis High intestinal iron concentrations due to low bioavailability cause gastrointestinal side effects (nausea, vomiting, abdominal pain and constipation) and limit compliance
Intravenous	Fast repletion of iron stores Safe if formulations with dextran are avoided Effective even when intestinal absorption is impaired	Higher expenses, including need for administration by a healthcare professional Potential risk for iron overload that in excess may contribute to oxidative stress Potential risk for anaphylactic reactions using dextran-containing formulations Hypophosphatemia with some preparations

* Anaemia of chronic disease (ACD).

## References

[1] Kassebaum N.J., Jasrasaria R., Naghavi M., Wulf S.K., Johns N., Lozano R., Regan M., Weatherall D., Chou D.P., Eisele T.P. (2014). A systematic analysis of global anemia burden from 1990 to 2010. Blood.

[2] Iron Deficinecy Anemia: Assessment, Prevention, and Control. A Guide for Programme Managers. http://www.who.int/nutrition/publications/en/ida_assessment_prevention_control.pdf.

[3] Gulmez H., Akin Y., Savas M., Gulum M., Ciftci H., Yalcinkaya S., Yeni E. (2014). Impact of iron supplementation on sexual dysfunction of women with iron deficiency anemia in short term: A preliminary study. J. Sex. Med..

[4] Haas J.D., Brownlie T. (2001). Iron deficiency and reduced work capacity: A critical review of the research to determine a causal relationship. J. Nutr..

[5] McClung J.P., Murray-Kolb L.E. (2013). Iron nutrition and premenopausal women: Effects of poor iron status on physical and neuropsychological performance. Annu. Rev. Nutr..

[6] Bresgen N., Eckl P.M. (2015). Oxidative stress and the homeodynamics of iron metabolism. Biomolecules.

[7] Filmann N., Rey J., Schneeweiss S., Ardizzone S., Bager P., Bergamaschi G., Koutroubakis I., Lindgren S., Morena F.L., Moum B. (2014). Prevalence of anemia in inflammatory bowel diseases in european countries: A systematic review and individual patient data meta-analysis. Inflamm. Bowel Dis..

[8] Fiorino G., Allocca M., Danese S. (2014). Commentary: Anaemia in inflammatory bowel disease—The most common and ignored extra intestinal manifestation. Aliment. Pharmacol. Ther..

[9] Ng S.C., Shi H.Y., Hamidi N., Underwood F.E., Tang W., Benchimol E.I., Panaccione R., Ghosh S., Wu J.C.Y., Chan F.K.L. (2018). Worldwide incidence and prevalence of inflammatory bowel disease in the 21st century: A systematic review of population-based studies. Lancet.

[10] Eriksson C., Cao Y., Rundquist S., Zhulina Y., Henriksson I., Montgomery S., Halfvarson J. (2017). Changes in medical management and colectomy rates: A population-based cohort study on the epidemiology and natural history of ulcerative colitis in Orebro, Sweden, 1963–2010. Aliment. Pharmacol. Ther..

[11] Molodecky N.A., Soon I.S., Rabi D.M., Ghali W.A., Ferris M., Chernoff G., Benchimol E.I., Panaccione R., Ghosh S., Barkema H.W. (2012). Increasing incidence and prevalence of the inflammatory bowel diseases with time, based on systematic review. Gastroenterology.

[12] Kaplan G.G., Jess T. (2016). The Changing Landscape of Inflammatory Bowel Disease: East Meets West. Gastroenterology.

[13] Portela F., Lago P., Cotter J., Goncalves R., Vasconcelos H., Ministro P., Lopes S., Eusebio M., Morna H., Cravo M. (2016). Anaemia in patients with inflammatory bowel disease—A nationwide cross-sectional study. Digestion.

[14] Hoivik M.L., Reinisch W., Cvancarova M., Moum B. (2014). Anaemia in inflammatory bowel disease: A population-based 10-year follow-up. Aliment. Pharmacol. Ther..

[15] Azzopardi N., Ellul P. (2014). Iron deficiency in Crohn’s disease: Iron supplementation or disease control?. J. Crohn’s Colitis.

[16] Bager P., Befrits R., Wikman O., Lindgren S., Moum B., Hjortswang H., Dahlerup J.F. (2013). High burden of iron deficiency and different types of anemia in inflammatory bowel disease outpatients in Scandinavia: A longitudinal 2-year follow-up study. Scand. J. Gastroenterol..

[17] Gisbert J.P., Gomollon F. (2008). Common misconceptions in the diagnosis and management of anemia in inflammatory bowel disease. Am. J. Gastroenterol..

[18] Larsen S., Bendtzen K., Nielsen O.H. (2010). Extraintestinal manifestations of inflammatory bowel disease: Epidemiology, diagnosis, and management. Ann. Med..

[19] Goodhand J.R., Kamperidis N., Rao A., Laskaratos F., McDermott A., Wahed M., Naik S., Croft N.M., Lindsay J.O., Sanderson I.R. (2012). Prevalence and management of anemia in children, adolescents, and adults with inflammatory bowel disease. Inflamm. Bowel Dis..

[20] Vagianos K., Clara I., Carr R., Graff L.A., Walker J.R., Targownik L.E., Lix L.M., Rogala L., Miller N., Bernstein C.N. (2015). What are adults with inflammatory bowel disease (IBD) eating? A closer look at the dietary habits of a population-based Canadian IBD cohort. J. Parenter. Enter. Nutr..

[21] Theurl I., Aigner E., Theurl M., Nairz M., Seifert M., Schroll A., Sonnweber T., Eberwein L., Witcher D.R., Murphy A.T. (2009). Regulation of iron homeostasis in anemia of chronic disease and iron deficiency anemia: Diagnostic and therapeutic implications. Blood.

[22] Weiss G., Gasche C. (2010). Pathogenesis and treatment of anemia in inflammatory bowel disease. Haematologica.

[23] Hwang C., Ross V., Mahadevan U. (2012). Micronutrient deficiencies in inflammatory bowel disease: From A to zinc. Inflamm. Bowel Dis..

[24] Murawska N., Fabisiak A., Fichna J. (2016). Anemia of chronic disease and iron deficiency anemia in inflammatory bowel diseases: Pathophysiology, diagnosis, and treatment. Inflamm. Bowel Dis..

[25] Gasche C., Berstad A., Befrits R., Beglinger C., Dignass A., Erichsen K., Gomollon F., Hjortswang H., Koutroubakis I., Kulnigg S. (2007). Guidelines on the diagnosis and management of iron deficiency and anemia in inflammatory bowel diseases. Inflamm. Bowel Dis..

[26] Goldberg N.D. (2013). Iron deficiency anemia in patients with inflammatory bowel disease. Clin. Exp. Gastroenterol..

[27] Semrin G., Fishman D.S., Bousvaros A., Zholudev A., Saunders A.C., Correia C.E., Nemeth E., Grand R.J., Weinstein D.A. (2006). Impaired intestinal iron absorption in Crohn’s disease correlates with disease activity and markers of inflammation. Inflamm. Bowel Dis..

[28] Weiss G., Goodnough L.T. (2005). Anemia of chronic disease. N. Engl. J. Med..

[29] Weiss G., Schett G. (2013). Anaemia in inflammatory rheumatic diseases. Nat. Rev. Rheumatol..

[30] Nairz M., Schroll A., Demetz E., Tancevski I., Theurl I., Weiss G. (2015). ‘Ride on the ferrous wheel’—The cycle of iron in macrophages in health and disease. Immunobiology.

[31] Camaschella C. (2015). Iron-deficiency anemia. N. Engl. J. Med..

[32] Shander A., Goodnough L.T., Javidroozi M., Auerbach M., Carson J., Ershler W.B., Ghiglione M., Glaspy J., Lew I. (2014). Iron deficiency anemia-bridging the knowledge and practice gap. Transfus. Med. Rev..

[33] Stein J., Hartmann F., Dignass A.U. (2010). Diagnosis and management of iron deficiency anemia in patients with IBD. Nat. Rev. Gastroenterol. Hepatol..

[34] Pizzi L.T., Weston C.M., Goldfarb N.I., Moretti D., Cobb N., Howell J.B., Infantolino A., Dimarino A.J., Cohen S. (2006). Impact of chronic conditions on quality of life in patients with inflammatory bowel disease. Inflamm. Bowel Dis..

[35] De Silva A.D., Tsironi E., Feakins R.M., Rampton D.S. (2005). Efficacy and tolerability of oral iron therapy in inflammatory bowel disease: A prospective, comparative trial. Aliment. Pharmacol. Ther..

[36] Bager P., Befrits R., Wikman O., Lindgren S., Moum B., Hjortswang H., Hjollund N.H., Dahlerup J.F. (2012). Fatigue in out-patients with inflammatory bowel disease is common and multifactorial. Aliment. Pharmacol. Ther..

[37] Gasche C., Lomer M.C., Cavill I., Weiss G. (2004). Iron, anaemia, and inflammatory bowel diseases. Gut.

[38] Dignass A.U., Gasche C., Bettenworth D., Birgegard G., Danese S., Gisbert J.P., Gomollon F., Iqbal T., Katsanos K., Koutroubakis I. (2015). European consensus on the diagnosis and management of iron deficiency and anaemia in inflammatory bowel diseases. J. Crohn’s Colitis.

[39] Martin J., Radeke H.H., Dignass A., Stein J. (2017). Current evaluation and management of anemia in patients with inflammatory bowel disease. Expert Rev. Gastroenterol. Hepatol..

[40] Stein J., Bager P., Befrits R., Gasche C., Gudehus M., Lerebours E., Magro F., Mearin F., Mitchell D., Oldenburg B. (2013). Anaemia management in patients with inflammatory bowel disease: Routine practice across nine European countries. Eur. J. Gastroenterol. Hepatol..

[41] Hentze M.W., Muckenthaler M.U., Galy B., Camaschella C. (2010). Two to tango: Regulation of Mammalian iron metabolism. Cell.

[42] Zhang C. (2014). Essential functions of iron-requiring proteins in DNA replication, repair and cell cycle control. Protein Cell.

[43] Pantopoulos K., Porwal S.K., Tartakoff A., Devireddy L. (2012). Mechanisms of mammalian iron homeostasis. Biochemistry.

[44] Coad J., Conlon C. (2011). Iron deficiency in women: Assessment, causes and consequences. Curr. Opin. Clin. Nutr. Metab. Care.

[45] Andrews N.C. (1999). Disorders of iron metabolism. N. Engl. J. Med..

[46] McDermid J.M., Lonnerdal B. (2012). Iron. Adv. Nutr..

[47] Hurrell R., Egli I. (2010). Iron bioavailability and dietary reference values. Am. J. Clin. Nutr..

[48] Ganz T., Nemeth E. (2012). Hepcidin and iron homeostasis. Biochim. Biophys. Acta.

[49] Nemeth E., Tuttle M.S., Powelson J., Vaughn M.B., Donovan A., Ward D.M., Ganz T., Kaplan J. (2004). Hepcidin regulates cellular iron efflux by binding to ferroportin and inducing its internalization. Science.

[50] Ruchala P., Nemeth E. (2014). The pathophysiology and pharmacology of hepcidin. Trends Pharmacol. Sci..

[51] Ganz T., Nemeth E. (2011). Hepcidin and disorders of iron metabolism. Annu. Rev. Med..

[52] Bregman D.B., Morris D., Koch T.A., He A., Goodnough L.T. (2013). Hepcidin levels predict nonresponsiveness to oral iron therapy in patients with iron deficiency anemia. Am. J. Hematol..

[53] Koskenkorva-Frank T.S., Weiss G., Koppenol W.H., Burckhardt S. (2013). The complex interplay of iron metabolism, reactive oxygen species, and reactive nitrogen species: Insights into the potential of various iron therapies to induce oxidative and nitrosative stress. Free Radic. Biol. Med..

[54] Carvalho L., Brait D., Vaz M., Lollo P., Morato P., Oesterreich S., Raposo J., Freitas K. (2017). Partially hydrolyzed guar gum increases ferroportin expression in the colon of anemic growing rats. Nutrients.

[55] Ludwiczek S., Aigner E., Theurl I., Weiss G. (2003). Cytokine-mediated regulation of iron transport in human monocytic cells. Blood.

[56] Munoz M., Garcia-Erce J.A., Remacha A.F. (2011). Disorders of iron metabolism. Part II: Iron deficiency and iron overload. J. Clin. Pathol..

[57] Theurl I., Schroll A., Sonnweber T., Nairz M., Theurl M., Willenbacher W., Eller K., Wolf D., Seifert M., Sun C.C. (2011). Pharmacologic inhibition of hepcidin expression reverses anemia of chronic inflammation in rats. Blood.

[58] Kautz L., Jung G., Valore E.V., Rivella S., Nemeth E., Ganz T. (2014). Identification of erythroferrone as an erythroid regulator of iron metabolism. Nat. Genet..

[59] Peyssonnaux C., Zinkernagel A.S., Schuepbach R.A., Rankin E., Vaulont S., Haase V.H., Nizet V., Johnson R.S. (2007). Regulation of iron homeostasis by the hypoxia-inducible transcription factors (HIFs). J. Clin. Investig..

[60] Sonnweber T., Nachbaur D., Schroll A., Nairz M., Seifert M., Demetz E., Haschka D., Mitterstiller A.M., Kleinsasser A., Burtscher M. (2014). Hypoxia induced downregulation of hepcidin is mediated by platelet derived growth factor BB. Gut.

[61] Tanno T., Bhanu N.V., Oneal P.A., Goh S.H., Staker P., Lee Y.T., Moroney J.W., Reed C.H., Luban N.L., Wang R.H. (2007). High levels of GDF15 in thalassemia suppress expression of the iron regulatory protein hepcidin. Nat. Med..

[62] Theurl I., Schroll A., Nairz M., Seifert M., Theurl M., Sonnweber T., Kulaksiz H., Weiss G. (2011). Pathways for the regulation of hepcidin expression in anemia of chronic disease and iron deficiency anemia in vivo. Haematologica.

[63] Lasocki S., Baron G., Driss F., Westerman M., Puy H., Boutron I., Beaumont C., Montravers P. (2010). Diagnostic accuracy of serum hepcidin for iron deficiency in critically ill patients with anemia. Intensive Care Med..

[64] Mullin G.E. (2012). Micronutrients and inflammatory bowel disease. Nutr. Clin. Pract..

[65] Thomas C., Thomas L. (2005). Anemia of chronic disease: Pathophysiology and laboratory diagnosis. Lab. Hematol..

[66] Oldenburg B., Koningsberger J.C., Van Berge Henegouwen G.P., Van Asbeck B.S., Marx J.J. (2001). Iron and inflammatory bowel disease. Aliment. Pharmacol. Ther..

[67] Theurl I., Mattle V., Seifert M., Mariani M., Marth C., Weiss G. (2006). Dysregulated monocyte iron homeostasis and erythropoietin formation in patients with anemia of chronic disease. Blood.

[68] Weiss G. (2015). Anemia of chronic disorders: New diagnostic tools and new treatment strategies. Semin. Hematol..

[69] Anker S.D., Comin C.J., Filippatos G., Willenheimer R., Dickstein K., Drexler H., Luscher T.F., Bart B., Banasiak W., Niegowska J. (2009). Ferric carboxymaltose in patients with heart failure and iron deficiency. N. Engl. J. Med..

[70] Jankowska E.A., Malyszko J., Ardehali H., Koc-Zorawska E., Banasiak W., von Haehling S., Macdougall I.C., Weiss G., McMurray J.J., Anker S.D. (2013). Iron status in patients with chronic heart failure. Eur. Heart J..

[71] Cook J.D. (2005). Diagnosis and management of iron-deficiency anaemia. Best Pract. Res. Clin. Haematol..

[72] Arosio P., Levi S. (2002). Ferritin, iron homeostasis, and oxidative damage. Free Radic. Biol. Med..

[73] Goddard A.F., McIntyre A.S., Scott B.B. (2000). Guidelines for the management of iron deficiency anaemia. Gut.

[74] Lipschitz D.A., Cook J.D., Finch C.A. (1974). A clinical evaluation of serum ferritin as an index of iron stores. N. Engl. J. Med..

[75] Mast A.E., Blinder M.A., Gronowski A.M., Chumley C., Scott M.G. (1998). Clinical utility of the soluble transferrin receptor and comparison with serum ferritin in several populations. Clin. Chem..

[76] Van Santen S., de Mast Q., Oosting J.D., van Ede A., Swinkels D.W., van der Ven A.J.A.M. (2014). Hematologic parameters predicting a response to oral iron therapy in chronic inflammation. Haematologica.

[77] Archer N.M., Brugnara C. (2015). Diagnosis of iron-deficient states. Crit. Rev. Clin. Lab. Sci..

[78] Punnonen K., Irjala K., Rajamaki A. (1997). Serum transferrin receptor and its ratio to serum ferritin in the diagnosis of iron deficiency. Blood.

[79] Tessitore N., Solero G.P., Lippi G., Bassi A., Faccini G.B., Bedogna V., Gammaro L., Brocco G., Restivo G., Bernich P. (2001). The role of iron status markers in predicting response to intravenous iron in haemodialysis patients on maintenance erythropoietin. Nephrol. Dial. Transplant..

[80] Brugnara C., Schiller B., Moran J. (2006). Reticulocyte hemoglobin equivalent (Ret He) and assessment of iron-deficient states. Clin. Lab. Haematol..

[81] Goodnough L.T., Nemeth E., Ganz T. (2010). Detection, evaluation, and management of iron-restricted erythropoiesis. Blood.

[82] Van Santen S., van Dongen-Lases E.C., de Vegt F., Laarakkers C.M.M., van Riel P.L.C.M., van Ede A.E., Swinkels D.W. (2011). Hepcidin and hemoglobin content parameters in the diagnosis of iron deficiency in rheumatoid arthritis patients with anemia. Arthritis Rheum..

[83] Girelli D., Nemeth E., Swinkels D.W. (2016). Hepcidin in the diagnosis of iron disorders. Blood.

[84] Prentice A.M., Doherty C.P., Abrams S.A., Cox S.E., Atkinson S.H., Verhoef H., Armitage A.E., Drakesmith H. (2012). Hepcidin is the major predictor of erythrocyte iron incorporation in anemic African children. Blood.

[85] Wilson A., Reyes E., Ofman J. (2004). Prevalence and outcomes of anemia in inflammatory bowel disease: A systematic review of the literature. Am. J. Med..

[86] Gomollon F., Gisbert J.P., Garcia-Erce J.A. (2010). Intravenous iron in digestive diseases: A clinical (re)view. Ther. Adv. Chronic Dis..

[87] Klein H.G., Spahn D.R., Carson J.L. (2007). Red blood cell transfusion in clinical practice. Lancet.

[88] Goodnough L.T., Bach R.G. (2001). Anemia, transfusion, and mortality. N. Engl. J. Med..

[89] Villanueva C., Colomo A., Bosch A., Concepcion M., Hernandez-Gea V., Aracil C., Graupera I., Poca M., Alvarez-Urturi C., Gordillo J. (2013). Transfusion strategies for acute upper gastrointestinal bleeding. N. Engl. J. Med..

[90] Taylor R.W., Manganaro L., O’Brien J., Trottier S.J., Parkar N., Veremakis C. (2002). Impact of allogenic packed red blood cell transfusion on nosocomial infection rates in the critically ill patient. Crit. Care Med..

[91] Talbot T.R., D’Agata E.M., Brinsko V., Lee B., Speroff T., Schaffner W. (2004). Perioperative blood transfusion is predictive of poststernotomy surgical site infection: Marker for morbidity or true immunosuppressant?. Clin. Infect. Dis..

[92] Aubron C., Nichol A., Cooper D.J., Bellomo R. (2013). Age of red blood cells and transfusion in critically ill patients. Ann. Intensive Care.

[93] Bihl F., Castelli D., Marincola F., Dodd R.Y., Brander C. (2007). Transfusion-transmitted infections. J. Transl. Med..

[94] Guinet F., Carniel E., Leclercq A. (2011). Transfusion-transmitted Yersinia enterocolitica sepsis. Clin. Infect. Dis..

[95] Cancelo-Hidalgo M.J., Castelo-Branco C., Palacios S., Haya-Palazuelos J., Ciria-Recasens M., Manasanch J., Perez-Edo L. (2013). Tolerability of different oral iron supplements: A systematic review. Curr. Med. Res. Opin..

[96] Santiago P. (2012). Ferrous versus ferric oral iron formulations for the treatment of iron deficiency: A clinical overview. Sci. World J..

[97] Fuqua B.K., Vulpe C.D., Anderson G.J. (2012). Intestinal iron absorption. J. Trace Elem. Med. Biol..

[98] Aspuru K., Villa C., Bermejo F., Herrero P., Lopez S.G. (2011). Optimal management of iron deficiency anemia due to poor dietary intake. Int. J. Gen. Med..

[99] Lane D.J., Richardson D.R. (2014). The active role of vitamin C in mammalian iron metabolism: Much more than just enhanced iron absorption!. Free Radic. Biol. Med..

[100] Syed S., Michalski E.S., Tangpricha V., Chesdachai S., Kumar A., Prince J., Ziegler T.R., Suchdev P.S., Kugathasan S. (2017). Vitamin D Status is associated with hepcidin and hemoglobin concentrations in children with inflammatory bowel disease. Inflamm. Bowel Dis..

[101] Mouli V.P., Ananthakrishnan A.N. (2014). Review article: Vitamin D and inflammatory bowel diseases. Aliment. Pharmacol. Ther..

[102] Bacchetta J., Zaritsky J.J., Sea J.L., Chun R.F., Lisse T.S., Zavala K., Nayak A., Wesseling-Perry K., Westerman M., Hollis B.W. (2014). Suppression of iron-regulatory hepcidin by vitamin D. J. Am. Soc. Nephrol..

[103] Gubatan J., Mitsuhashi S., Zenlea T., Rosenberg L., Robson S., Moss A.C. (2017). Low serum vitamin D during remission increases risk of clinical relapse in patients with ulcerative colitis. Clin. Gastroenterol. Hepatol..

[104] Kabbani T.A., Koutroubakis I.E., Schoen R.E., Ramos-Rivers C., Shah N., Swoger J., Regueiro M., Barrie A., Schwartz M., Hashash J.G. (2016). Association of vitamin D level with clinical status in inflammatory bowel disease: A 5-year longitudinal study. Am. J. Gastroenterol..

[105] Winter R.W., Collins E., Cao B., Carrellas M., Crowell A.M., Korzenik J.R. (2017). Higher 25-hydroxyvitamin D levels are associated with greater odds of remission with anti-tumour necrosis factor-alpha medications among patients with inflammatory bowel diseases. Aliment. Pharmacol. Ther..

[106] Smith E.M., Tangpricha V. (2015). Vitamin D and anemia: Insights into an emerging association. Curr. Opin. Endocrinol. Diabetes Obes..

[107] Smith E.M., Jones J.L., Han J.E., Alvarez J.A., Sloan J.H., Konrad R.J., Zughaier S.M., Martin G.S., Ziegler T.R., Tangpricha V. (2018). High-dose vitamin D_3_ administration is associated with increases in hemoglobin concentrations in mechanically ventilated critically III adults: A pilot double-blind, randomized, placebo-controlled trial. J. Parenter. Enter. Nutr..

[108] Zughaier S.M., Alvarez J.A., Sloan J.H., Konrad R.J., Tangpricha V. (2014). The role of vitamin D in regulating the iron-hepcidin-ferroportin axis in monocytes. J. Clin. Transl. Endocrinol..

[109] Smith E.M., Alvarez J.A., Kearns M.D., Hao L., Sloan J.H., Konrad R.J., Ziegler T.R., Zughaier S.M., Tangpricha V. (2017). High-dose vitamin D_3_ reduces circulating hepcidin concentrations: A pilot, randomized, double-blind, placebo-controlled trial in healthy adults. Clin. Nutr..

[110] Gasche C., Ahmad T., Tulassay Z., Baumgart D.C., Bokemeyer B., Buning C., Howaldt S., Stallmach A. (2015). Ferric maltol is effective in correcting iron deficiency anemia in patients with inflammatory bowel disease: Results from a phase-3 clinical trial program. Inflamm. Bowel Dis..

[111] Hallberg L., Ryttinger L., Solvell L. (1966). Side-effects of oral iron therapy. A double-blind study of different iron compounds in tablet form. J. Intern. Med..

[112] Moretti D., Goede J.S., Zeder C., Jiskra M., Chatzinakou V., Tjalsma H., Melse-Boonstra A., Brittenham G., Swinkels D.W., Zimmermann M.B. (2015). Oral iron supplements increase hepcidin and decrease iron absorption from daily or twice-daily doses in iron-depleted young women. Blood.

[113] Stoffel N.U., Cercamondi C.I., Brittenham G., Zeder C., Geurts-Moespot A.J., Swinkels D.W., Moretti D., Zimmermann M.B. (2017). Iron absorption from oral iron supplements given on consecutive versus alternate days and as single morning doses versus twice-daily split dosing in iron-depleted women: Two open-label, randomised controlled trials. Lancet Haematol..

[114] Kulnigg S., Gasche C. (2006). Systematic review: Managing anaemia in Crohn’s disease. Aliment. Pharmacol. Ther..

[115] Iqbal T., Stein J., Sharma N., Kulnigg-Dabsch S., Vel S., Gasche C. (2015). Clinical significance of C-reactive protein levels in predicting responsiveness to iron therapy in patients with inflammatory bowel disease and iron deficiency anemia. Dig. Dis. Sci..

[116] Erichsen K., Milde A.M., Arslan G., Helgeland L., Gudbrandsen O.A., Ulvik R.J., Berge R.K., Hausken T., Berstad A. (2005). Low-dose oral ferrous fumarate aggravated intestinal inflammation in rats with DSS-induced colitis. Inflamm. Bowel Dis..

[117] Rizvi S., Schoen R.E. (2011). Supplementation with oral vs. intravenous iron for anemia with IBD or gastrointestinal bleeding: Is oral iron getting a bad rap?. Am. J. Gastroenterol..

[118] Lee T., Clavel T., Smirnov K., Schmidt A., Lagkouvardos I., Walker A., Lucio M., Michalke B., Schmitt-Kopplin P., Fedorak R. (2017). Oral versus intravenous iron replacement therapy distinctly alters the gut microbiota and metabolome in patients with IBD. Gut.

[119] Lee T.W., Kolber M.R., Fedorak R.N., van Zanten S.V. (2012). Iron replacement therapy in inflammatory bowel disease patients with iron deficiency anemia: A systematic review and meta-analysis. J. Crohn’s Colitis.

[120] Kostic A.D., Xavier R.J., Gevers D. (2014). The microbiome in inflammatory bowel disease: Current status and the future ahead. Gastroenterology.

[121] Werner T., Wagner S.J., Martinez I., Walter J., Chang J.S., Clavel T., Kisling S., Schuemann K., Haller D. (2011). Depletion of luminal iron alters the gut microbiota and prevents Crohn’s disease-like ileitis. Gut.

[122] Kulnigg S., Teischinger L., Dejaco C., Waldhor T., Gasche C. (2009). Rapid recurrence of IBD-associated anemia and iron deficiency after intravenous iron sucrose and erythropoietin treatment. Am. J. Gastroenterol..

[123] Vadhan-Raj S., Strauss W., Ford D., Bernard K., Boccia R., Li J., Allen L.F. (2014). Efficacy and safety of IV ferumoxytol for adults with iron deficiency anemia previously unresponsive to or unable to tolerate oral iron. Am. J. Hematol..

[124] New recommendations to manage risk of allergic reactions with intravenous iron-containing Medicines. http://www.ema.europa.eu/ema/index.jsp?curl=pages/news_and_events/news/2013/06/news_detail_001833.jsp&mid=WC0b01ac058004d5c1.

[125] Highlights of prescription information. http://www.accessdata.fda.gov/drugsatfda_docs/label/2013/203565s000lbl.pdf.

[126] Auerbach M., Rodgers G.M. (2007). Intravenous iron. N. Engl. J. Med..

[127] Rampton D., Folkersen J., Fishbane S., Hedenus M., Howaldt S., Locatelli F., Patni S., Szebeni J., Weiss G. (2014). Hypersensitivity reactions to intravenous iron: Guidance for risk minimization and management. Haematologia.

[128] Gisbert J.P., Bermejo F., Pajares R., Perez-Calle J.L., Rodriguez M., Algaba A., Mancenido N., de la Morena F., Carneros J.A., McNicholl A.G. (2009). Oral and intravenous iron treatment in inflammatory bowel disease: Hematological response and quality of life improvement. Inflamm. Bowel Dis..

[129] Reinisch W., Staun M., Tandon R.K., Altorjay I., Thillainayagam A.V., Gratzer C., Nijhawan S., Thomsen L.L. (2013). A randomized, open-label, non-inferiority study of intravenous iron isomaltoside 1000 (Monofer) compared with oral iron for treatment of anemia in IBD (PROCEED). Am. J. Gastroenterol..

[130] Auerbach M., Ballard H. (2010). Clinical use of intravenous iron: Administration, efficacy, and safety. Hematology. ASH Educ. Program Book.

[131] Gomollon F., Gisbert J.P. (2013). Intravenous iron in inflammatory bowel diseases. Curr. Opin. Gastroenterol..

[132] Gomollon F., Chowers Y., Danese S., Dignass A., Nielsen O.H., Lakatos P.L., Lees C.W., Lindgren S., Lukas M., Mantzaris G.J. (2014). Letter: European Medicines Agency recommendations for allergic reactions to intravenous iron-containing medicines. Aliment. Pharmacol. Ther..

[133] Chertow G.M., Mason P.D., Vaage-Nilsen O., Ahlmen J. (2006). Update on adverse drug events associated with parenteral iron. Nephrol. Dial. Transplant..

[134] Khalil A., Goodhand J.R., Wahed M., Yeung J., Ali F.R., Rampton D.S. (2011). Efficacy and tolerability of intravenous iron dextran and oral iron in inflammatory bowel disease: A case-matched study in clinical practice. Eur. J. Gastroenterol. Hepatol..

[135] Koutroubakis I.E., Oustamanolakis P., Karakoidas C., Mantzaris G.J., Kouroumalis E.A. (2010). Safety and efficacy of total-dose infusion of low molecular weight iron dextran for iron deficiency anemia in patients with inflammatory bowel disease. Dig. Dis. Sci..

[136] Rodgers G.M., Auerbach M., Cella D., Chertow G.M., Coyne D.W., Glaspy J.A., Henry D.H. (2008). High-molecular weight iron dextran: A wolf in sheep’s clothing?. J. Am. Soc. Nephrol..

[137] Auerbach M., Pappadakis J.A., Bahrain H., Auerbach S.A., Ballard H., Dahl N.V. (2011). Safety and efficacy of rapidly administered (one hour) one gram of low molecular weight iron dextran (INFeD) for the treatment of iron deficient anemia. Am. J. Hematol..

[138] Schroder O., Mickisch O., Seidler U., de Weerth A., Dignass A.U., Herfarth H., Reinshagen M., Schreiber S., Junge U., Schrott M. (2005). Intravenous iron sucrose versus oral iron supplementation for the treatment of iron deficiency anemia in patients with inflammatory bowel disease—A randomized, controlled, open-label, multicenter study. Am. J. Gastroenterol..

[139] Reed J. (2010). Reed Book: Pharmacy’s Fundamental Reference.

[140] Esposito B.P., Breuer W., Sirankapracha P., Pootrakul P., Hershko C., Cabantchik Z.I. (2003). Labile plasma iron in iron overload: Redox activity and susceptibility to chelation. Blood.

[141] Beigel F., Lohr B., Laubender R.P., Tillack C., Schnitzler F., Breiteneicher S., Weidinger M., Goke B., Seiderer J., Ochsenkuhn T. (2012). Iron status and analysis of efficacy and safety of ferric carboxymaltose treatment in patients with inflammatory bowel disease. Digestion.

[142] Evstatiev R., Marteau P., Iqbal T., Khalif I.L., Stein J., Bokemeyer B., Chopey I.V., Gutzwiller F.S., Riopel L., Gasche C. (2011). FERGIcor, a randomized controlled trial on ferric carboxymaltose for iron deficiency anemia in inflammatory bowel disease. Gastroenterology.

[143] Evstatiev R., Alexeeva O., Bokemeyer B., Chopey I., Felder M., Gudehus M., Iqbal T., Khalif I., Marteau P., Stein J. (2013). Ferric carboxymaltose prevents recurrence of anemia in patients with inflammatory bowel disease. Clin. Gastroenterol. Hepatol..

[144] Kulnigg S., Stoinov S., Simanenkov V., Dudar L.V., Karnafel W., Garcia L.C., Sambuelli A.M., D’Haens G., Gasche C. (2008). A novel intravenous iron formulation for treatment of anemia in inflammatory bowel disease: The ferric carboxymaltose (FERINJECT) randomized controlled trial. Am. J. Gastroenterol..

[145] Onken J.E., Bregman D.B., Harrington R.A., Morris D., Acs P., Akright B., Barish C., Bhaskar B.S., Smith-Nguyen G.N., Butcher A. (2014). A multicenter, randomized, active-controlled study to investigate the efficacy and safety of intravenous ferric carboxymaltose in patients with iron deficiency anemia. Transfusion.

[146] Gozzard D. (2011). When is high-dose intravenous iron repletion needed? Assessing new treatment options. Drug Des. Dev. Ther..

[147] Nordfjeld K., Andreasen H., Thomsen L.L. (2012). Pharmacokinetics of iron isomaltoside 1000 in patients with inflammatory bowel disease. Drug Des. Dev. Ther..

[148] Auerbach M., Strauss W., Auerbach S., Rineer S., Bahrain H. (2013). Safety and efficacy of total dose infusion of 1020 mg of ferumoxytol administered over 15 min. Am. J. Hematol..

[149] Ford D.C., Dahl N.V., Strauss W.E., Barish C.F., Hetzel D.J., Bernard K., Li Z., Allen L.F. (2016). Ferumoxytol versus placebo in iron deficiency anemia: Efficacy, safety, and quality of life in patients with gastrointestinal disorders. Clin. Exp. Gastroenterol..

[150] Schieda N. (2013). Parenteral ferumoxytol interaction with magnetic resonance imaging: A case report, review of the literature and advisory warning. Insights Imaging.

[151] Bailie G.R. (2012). Comparison of rates of reported adverse events associated with i.v. iron products in the United States. Am. J. Health Syst. Pharm..

[152] Szebeni J., Fishbane S., Hedenus M., Howaldt S., Locatelli F., Patni S., Rampton D., Weiss G., Folkersen J. (2015). Hypersensitivity to intravenous iron: Classification, terminology, mechanisms and management. Br. J. Pharmacol..

[153] Auerbach M., Ballard H., Glaspy J. (2007). Clinical update: Intravenous iron for anaemia. Lancet.

[154] Chertow G.M., Winkelmayer W.C. (2010). On the relative safety of intravenous iron formulations: New answers, new questions. Am. J. Hematol..

[155] Fishbane S., Ungureanu V.D., Maesaka J.K., Kaupke C.J., Lim V., Wish J. (1996). The safety of intravenous iron dextran in hemodialysis patients. Am. J. Kidney Dis..

[156] Auerbach M., Coyne D., Ballard H. (2008). Intravenous iron: From anathema to standard of care. Am. J. Hematol..

[157] Boyce M., Warrington S., Cortezi B., Zollner S., Vauleon S., Swinkels D.W., Summo L., Schwoebel F., Riecke K. (2016). Safety, pharmacokinetics and pharmacodynamics of the anti-hepcidin Spiegelmer Lexaptepid pegol in healthy subjects. Br. J. Pharmacol..

[158] Cooke K.S., Hinkle B., Salimi-Moosavi H., Foltz I., King C., Rathanaswami P., Winters A., Steavenson S., Begley C.G., Molineux G. (2013). A fully human anti-hepcidin antibody modulates iron metabolism in both mice and nonhuman primates. Blood.

[159] Sebastiani G., Wilkinson N., Pantopoulos K. (2016). Pharmacological targeting of the hepcidin/ferroportin axis. Front. Pharmacol..

[160] Sun C.C., Vaja V., Chen S., Theurl I., Stepanek A., Brown D.E., Cappellini M.D., Weiss G., Hong C.C., Lin H.Y. (2013). A hepcidin lowering agent mobilizes iron for incorporation into red blood cells in an adenine-induced kidney disease model of anemia in rats. Nephrol. Dial. Transplant..

[161] Gupta N., Wish J.B. (2017). Hypoxia-inducible factor prolyl hydroxylase inhibitors: A potential new treatment for anemia in patients with CKD. Am. J. Kidney Dis..

[162] Haase V.H. (2017). HIF-prolyl hydroxylases as therapeutic targets in erythropoiesis and iron metabolism. Hemodial. Int..

[163] Simpson R.J., McKie A.T. (2015). Iron and oxygen sensing: A tale of 2 interacting elements?. Metallomics.

[164] Nielsen O.H., Ainsworth M., Coskun M., Weiss G. (2015). Management of iron-deficiency anemia in inflammatory bowel disease: A systematic review. Medicine.

[165] Lindgren S., Wikman O., Befrits R., Blom H., Eriksson A., Granno C., Ung K.A., Hjortswang H., Lindgren A., Unge P. (2009). Intravenous iron sucrose is superior to oral iron sulphate for correcting anaemia and restoring iron stores in IBD patients: A randomized, controlled, evaluator-blind, multicentre study. Scand. J. Gastroenterol..

[166] Litton E., Xiao J., Ho K.M. (2013). Safety and efficacy of intravenous iron therapy in reducing requirement for allogeneic blood transfusion: Systematic review and meta-analysis of randomised clinical trials. BMJ.

[167] Fishbane S. (2016). Balance of benefit and risk in intravenous iron treatment in chronic kidney disease. Semin. Nephrol..

[168] Li X., Kshirsagar A.V., Brookhart M.A. (2017). Safety of intravenous iron in hemodialysis patients. Hemodial. Int..

[169] Macdougall I.C., Bircher A.J., Eckardt K.U., Obrador G.T., Pollock C.A., Stenvinkel P., Swinkels D.W., Wanner C., Weiss G., Chertow G.M. (2016). Iron management in chronic kidney disease: Conclusions from a “Kidney Disease: Improving Global Outcomes” (KDIGO) Controversies Conference. Kidney Int..

[170] Miskulin D.C., Tangri N., Bandeen-Roche K., Zhou J., McDermott A., Meyer K.B., Ephraim P.L., Michels W.M., Jaar B.G., Crews D.C. (2014). Intravenous iron exposure and mortality in patients on hemodialysis. Clin. J. Am. Soc. Nephrol..

[171] Zitt E., Sturm G., Kronenberg F., Neyer U., Knoll F., Lhotta K., Weiss G. (2014). Iron supplementation and mortality in incident dialysis patients: An observational study. PLoS ONE.

[172] Aksan A., Isik H., Radeke H.H., Dignass A., Stein J. (2017). Systematic review with network meta-analysis: Comparative efficacy and tolerability of different intravenous iron formulations for the treatment of iron deficiency anaemia in patients with inflammatory bowel disease. Aliment. Pharmacol. Ther..

[173] Gonzalez A.C., Pedrajas C.C., Marin P.S., Benitez J.M., Iglesias F.E., Salgueiro R., Medina M.R., Garcia-Sanchez V. (2017). Prevalence of iron deficiency without anaemia in inflammatory bowel disease and impact on health-related quality of life. Gastroenterol. Hepatol..

[174] Krayenbuehl P.A., Battegay E., Breymann C., Furrer J., Schulthess G. (2011). Intravenous iron for the treatment of fatigue in nonanemic, premenopausal women with low serum ferritin concentration. Blood.

[175] Cekic C., Iepk S., Aslan F., Akpinat Z., Arabul M., Topal F., Saritas-Yüksel E., Alper E., Ünsal B. (2015). The effect of intravenous iron treatment on quality of life in inflammatory bowel disease patients with nonanemic iron deficiency. Gastroenterol. Res. Pract..

[176] Eliadou E., Kini G., Huang J., Champion A., Inns S.J. (2017). Intrevenous iron replacement improves quality of life in hypoferritinemic inflammatory bowel disease patients with and without anemia. Dig. Dis..

[177] Favrat B., Balck K., Breymann C., Hedenus M., Keller T., Mezzacasa A., Gasche C. (2014). Evaluation of a single dose of ferric carboxymaltose in fatigued, iron-deficient women—PREFER a randomized, placebo-controlled study. PLoS ONE.

[178] Volani C., Doerrier C., Demetz E., Haschka D., Paglia G., Lavdas A.A., Gnaiger E., Weiss G. (2017). Dietary iron loading negatively affects liver mitochondrial function. Metallomics.

[179] Nielsen O.H., Coskun M., Weiss G. (2016). Iron replacement therapy: Do we need new guidelines?. Curr. Opin. Gastroenterol..

[180] Rimon E., Kagansky N., Kagansky M., Mechnick L., Mashiah T., Namir M., Levy S. (2005). Are we giving too much iron? Low-dose iron therapy is effective in octogenarians. Am. J. Med..

[181] Ganzoni A.M. (1970). Intravenous iron-dextran: Therapeutic and experimental possibilities. Schweiz. Med. Wochenschr..

[182] Reinisch W., Chowers Y., Danese S., Dignass A., Gomollon F., Nielsen O.H., Lakatos P.L., Lees C.W., Lindgren S., Lukas M. (2013). The management of iron deficiency in inflammatory bowel disease—An online tool developed by the RAND/UCLA appropriateness method. Aliment. Pharmacol. Ther..

[183] Katsanos K.H., Tatsioni A., Natsi D., Sigounas D., Christodoulou D.K., Tsianos E.V. (2012). Recombinant human erythropoietin in patients with inflammatory bowel disease and refractory anemia: A 15-year single center experience. J. Crohn’s Colitis.

[184] Liu S., Ren J., Hong Z., Yan D., Gu G., Han G., Wang G., Ren H., Chen J., Li J. (2013). Efficacy of erythropoietin combined with enteral nutrition for the treatment of anemia in Crohn’s disease: A prospective cohort study. Nutr. Clin. Pract..

[185] Solomon S.D., Uno H., Lewis E.F., Eckardt K.U., Lin J., Burdmann E.A., de Zeeuw D., Ivanovich P., Levey A.S., Parfrey P. (2010). Erythropoietic response and outcomes in kidney disease and type 2 diabetes. N. Engl. J. Med..

[186] Drueke T.B., Parfrey P.S. (2012). Summary of the KDIGO guideline on anemia and comment: Reading between the (guide)line(s). Kidney Int..

[187] Sonnweber T., Theurl I., Seifert M., Schroll A., Eder S., Mayer G., Weiss G. (2011). Impact of iron treatment on immune effector function and cellular iron status of circulating monocytes in dialysis patients. Nephrol. Dial. Transplant..

[188] Weiss G., Carver P.L. (2018). Role of divalent metals in infectious disease susceptibility and outcome. Clin. Microbiol. Infect..

[189] Sazawal S., Black R.E., Ramsan M., Chwaya H.M., Stoltzfus R.J., Dutta A., Dhingra U., Kabole I., Deb S., Othman M.K. (2006). Effects of routine prophylactic supplementation with iron and folic acid on admission to hospital and mortality in preschool children in a high malaria transmission setting: Community-based, randomised, placebo-controlled trial. Lancet.

[190] Soofi S., Cousens S., Iqbal S.P., Akhund T., Khan J., Ahmed I., Zaidi A.K., Bhutta Z.A. (2013). Effect of provision of daily zinc and iron with several micronutrients on growth and morbidity among young children in Pakistan: A cluster-randomised trial. Lancet.

[191] Besarab A., Bolton W.K., Browne J.K., Egrie J.C., Nissenson A.R., Okamoto D.M., Schwab S.J., Goodkin D.A. (1998). The effects of normal as compared with low hematocrit values in patients with cardiac disease who are receiving hemodialysis and epoetin. N. Engl. J. Med..

[192] Locatelli F., Pisoni R.L., Combe C., Bommer J., Andreucci V.E., Piera L., Greenwood R., Feldman H.I., Port F.K., Held P.J. (2004). Anaemia in haemodialysis patients of five European countries: Association with morbidity and mortality in the Dialysis Outcomes and Practice Patterns Study (DOPPS). Nephrol. Dial. Transplant..

[193] Bircher A.J., Auerbach M. (2014). Hypersensitivity from intravenous iron products. Immunol. Allergy Clin..

[194] Schaefer B., Wurtinger P., Finkenstedt A., Braithwaite V., Viveiros A., Effenberger M., Sulzbacher I., Moschen A., Griesmacher A., Tilg H. (2016). Choice of high-dose intravenous iron preparation determines hypophosphatemia risk. PLoS ONE.

[195] Goldsmith J.R., Sartor R.B. (2014). The role of diet on intestinal microbiota metabolism: Downstream impacts on host immune function and health, and therapeutic implications. J. Gastroenterol..

[196] Oustamanolakis P., Koutroubakis I.E., Messaritakis I., Malliaraki N., Sfiridaki A., Kouroumalis E.A. (2011). Serum hepcidin and prohepcidin concentrations in inflammatory bowel disease. Eur. J. Gastroenterol. Hepatol..

[197] Ganz T. (2013). Systemic iron homeostasis. Physiol. Rev..

[198] Katsanos K., Cavalier E., Ferrante M., Van Hauwaert V., Henckaerts L., Schnitzler F., Katsaraki A., Noman M., Vermeire S., Tsianos E.V. (2007). Intravenous iron therapy restores functional iron deficiency induced by infliximab. J. Crohn’s Colitis.

[199] Nielsen O.H., Ainsworth M.A. (2013). Tumor necrosis factor inhibitors for inflammatory bowel disease. N. Engl. J. Med..

